# Risk factors for radioactive iodine-avid metastatic lymph nodes on post I-131 ablation SPECT/CT in low- or intermediate-risk groups of papillary thyroid cancer

**DOI:** 10.1371/journal.pone.0202644

**Published:** 2018-08-17

**Authors:** Chang-Hee Lee, Ji-hoon Jung, Seung Hyun Son, Chae Moon Hong, Ju Hye Jeong, Shin Young Jeong, Sang-Woo Lee, Jaetae Lee, Byeong-Cheol Ahn

**Affiliations:** 1 Department of Nuclear Medicine, School of Medicine, Kyungpook National University, Daegu, South Korea; 2 Department of Nuclear Medicine, Kyungpook National University Hospital, Daegu, South Korea; Universidade Federal do Rio de Janeiro, BRAZIL

## Abstract

**Objective:**

Post I-131 ablation single-photon emission computed tomography (SPECT)/CT can show radioactive iodine (RAI)-avid cervical metastatic lymph nodes (mLN) in differentiated thyroid cancer. This study aimed to evaluate the incidence of RAI-avid mLN on post I-131 ablation SPECT/CT and the risk factors related to metastasis among patients with papillary thyroid cancer (PTC) in the low- or intermediate-risk groups.

**Study design and setting:**

Among 339 patients with PTC who underwent total thyroidectomy followed by I-131 ablation, 292 (228 women, 64 men) belonging to the low- or intermediate-risk groups before I-131 ablation, and with sufficient clinical follow-up data were enrolled. The risk groups were classified based on the American Thyroid Association 2015 guideline. Each patient was followed-up for at least 24 months after the ablation (median: 30 months). The clinical, pathologic, and biochemical factors of PTC were reviewed, and their relationships to RAI-avid mLN on SPECT/CT were analyzed.

**Results:**

Of the 292 patients, 61 and 231 belonged to the low-and intermediate-risk groups, respectively. Four (6.5%) patients in the low-risk group and 31 (13.0%) patients in the intermediate-risk group had RAI-avid mLN. A high preablation TSH-stimulated serum thyroglobulin (Tg) level in the low- or intermediate-risk group predicted the presence of RAI-avid mLN (cut-off = 0.5; hazard ratio (HR): 2.96; p = 0.04). In the subgroup analysis by risk group, TSH-stimulated serum Tg only predicted RAI-avid mLN in the low-risk group (cut-off = 1.0; HR: 5.3; p = 0.03).

**Conclusion:**

The incidence of RAI-avid mLN on postablation SPECT/CT was relatively high in both low- and intermediate-risk patients with PTC, and high preablation TSH-stimulated serum Tg level was a predictor of metastasis, especially in the low-risk group. A selective treatment approach should be considered in patients with high preablation TSH-stimulated serum Tg level.

## Introduction

Residual normal or malignant thyroid tissue after total thyroidectomy for differentiated thyroid carcinoma (DTC) is a common finding. Radioactive iodine (RAI) can be administered to thyroidectomized patients for remnant ablation or as an adjuvant therapy. Some retrospective studies have shown that RAI treatment improves the survival rate and decreases the recurrence rate of papillary thyroid cancer (PTC) [1, 2].

However, the 2015 American Thyroid Association (ATA) Management Guidelines for DTC do not recommend routine RAI remnant ablation for low-risk DTC patients [3]. The incidence of residual lymph node (LN) metastasis after total thyroidectomy is relatively high even in DTC patients with small intrathyroidal tumors [4]. Non-resected persistent LN metastasis can be found later and it is generally considered as a recurrence which contributes negatively to the prognosis of patients with DTC.

The introduction of single-photon emission computed tomography (SPECT)/CT technology has increased the diagnostic value of RAI scintigraphy. SPECT/CT can detect LN metastasis more sensitively and more specifically than a planar scan [4–6] and can facilitate accurate staging of thyroid cancer, which is helpful for establishing further treatment plans [7]. Because ultrasonography (US) is almost always performed preoperatively, the residual LN metastasis found on SPECT/CT after RAI ablation can be assumed to be not distinguishable via US. These residual lesions cause persistence or recurrence if not treated with RAI ablation. The 2015 ATA guideline does not recommend RAI administration for patients in the low-risk group; thus, possible residual LN metastases in these patients are less likely to be detected and treated until the development of metastatic LN (mLN). RAI administration is also not recommended for patients who belong to the intermediate-risk group; thus, persistent mLN in these patients will cause recurrence. Therefore, identifying predictive factors for the existence of RAI-avid mLN on postablation SPECT/CT (RxSPECT/CT) that can be assessed before deciding on RAI ablation in patients in the low- and intermediate-risk groups is important for individualized clinical management.

The current study aimed to evaluate the incidence of hidden RAI-avid mLN on RxSPECT/CT after curative surgery in patients with PTC belonging to the low- or intermediate-risk groups according to the 2015 ATA guideline, and to investigate the risk factors related to the occurrence of metastasis.

## Materials and methods

### Participants

This study involved 339 patients with PTC who underwent total thyroidectomy followed by RAI ablation in our hospital between September 2013 and December 2014. The patients who belonged to the low- or intermediate-risk group before RAI ablation were enrolled [3]. The risk groups were determined following the 2015 ATA guidelines. The low-risk group was defined as having no distant metastases, no tumor invasion of locoregional tissues or structures, no vascular invasion, no clinical evidence of LN metastases (cN0) on preoperative US and fewer than 5 pathologically involved neck LNs (pN1) with metastases, and a tumor that did not have an aggressive histology. The intermediate-risk group was defined as having microscopic extrathyroidal extension, cervical LN metastases, vascular invasion or aggressive tumor histology, clinical N1, or over 5 pN1. All patients performed preoperative ultrasonography (US) by an experienced radiologist, and fine-needle aspiration cytology was performed for suspicious regional LNs greater than 5–8 mm in the smallest diameter according to the ATA guidelines [3]. Most patients were diagnosed with PTC by preoperative US guided fine-needle aspiration cytology. Surgery was performed by two experienced high-volume thyroid surgeons who perform more than 300 cases of thyroid cancer surgery per year. All patients underwent total thyroidectomy, and prophylactic central compartment node dissection was also performed. In patients who had LN metastasis, therapeutic central and/or lateral neck node dissections were performed according to the extent of the LN metastasis. The diagnosis of PTC was reconfirmed for all patients by surgical pathology. If there is information about the size of mLN, the patients with LN with a diameter of 3 cm or more suggesting high risk group were excluded. Each patient was followed-up for at least 24 months after the RAI ablation (median: 30 months). Patients who had limited clinical information, with interfering anti-Tg antibodies (TgAb), or who were classified into the high-risk group according to the 2015 ATA guidelines [3] were excluded from the study. Data on the clinical (age, sex, and type of surgery) and pathological parameters (tumor size, number of mLN, ratio of mLN, pathologic TNM stage, and preablation thyroid-stimulating hormone (TSH)-stimulated serum Tg) were obtained from patient records.

### Ethics statement

The survey was approved by the institutional review board (IRB) of Kyungpook National University School of Medicine/Hospital, Korea. The patient records and information was anonymized and de-identified prior to analysis and all data was fully anonymized prior to access by any of the authors.

### RAI ablation and postablation scintigraphy with SPECT/CT

RAI ablation was performed at a median interval of 7 weeks (range: 4–14 weeks) after operation, either after the injection of recombinant human TSH (rhTSH; Thyrogen, Genzyme Corp., Cambridge, MA, USA) or after the withdrawal of thyroid hormone replacement (THW). Depending on the pathological result, the administered doses of I-131 ranged from 1.11 GBq to 5.55 GBq (30–150 mCi), and postablation I-131 scintigraphy was performed 4 to 5 days after treatment using a dual-head gamma camera equipped with 1.5875 cm NaI crystals and a multidetector (16-row) spiral CT (NM670; General Electric Medical Systems, Milwaukee, WI, USA). Post RAI ablation whole-body planar scan (RxWBS) was initially acquired at a scanning speed of 10 cm/min, after which SPECT/CT was performed. A 128 × 128 matrix was used, and 6430-sec projections were acquired over 360 degrees for the SPECT acquisition. SPECT data were reconstructed using a 3-dimensional iterative algorithm. CT scans were obtained at a tube voltage of 120 kV. The slice width and spacing were 3.75 mm. CT data were used for anatomical information and attenuation correction. Data of RxWBS and SPECT/CT were analyzed with Xeleris software (General Electric Medical Systems, Milwaukee, WI, USA). No contrast medium was used during the procedure.

### Laboratory studies

We measured serum TSH, Tg, and TgAb immediately before RAI administration. The serum Tg and serum TgAb levels were evaluated using Thyroglobulin IRMA (Cis Bio International, Gif-sur-Yvette, France) and Tg Antibody Coated Tube Assay (RSR, Cardiff, UK) kits. Meanwhile, serum TSH (TSH IRMA; BRAHMS, Hennigsdorf, Germany) was measured via immunoradiometric assay commercial kits. TSH levels > 30 μU/mL were considered adequate to increase RAI uptake in thyroid tumor cells during RAI ablation.

### Data analysis

All images from RxWBS and RxSPECT/CT were analyzed separately by two experienced nuclear medicine physicians. On RxWBS, RAI uptake in the esophageal tract and salivary glands were considered to be physiological, and other focal tracer uptake on the background that was not compatible with the residual thyroid tissue or physiological activity was considered as a pathologic uptake. The SPECT/CT images were classified as (a) remnant tissue if the median foci were localized either in the upper thyroid bed, in the thyroglossal duct remnants, or in the lower thyroid bed or (b) LN metastasis if the focus was localized in front of a round lesion identical to an LN.

### Statistical methods

Continuous data are presented as medians and ranges or means and standard deviations, as appropriate for each variable. Chi-square tests were performed for categorical comparisons, and continuous variables were compared using Mann–Whitney *U*-test. To identify the optimal cut-off for serum Tg level for predicting RAI-avid mLN on RxSPECT/CT, receiver operating characteristic (ROC) curve analysis was used. Logistic regression was used for the multivariate analysis. MedCalc Statistical Software version 13.2 was used for all statistical calculations, and p values < 0.05 were considered statistically significant.

## Results

### Patient characteristics

A total of 292 patients (228 women, 64 men; average age: 48.5 ± 12.5 years) belonged to the low- (N = 61) or intermediate-risk (N = 231) groups before RAI ablation. The demographic characteristics of the patients are summarized in Table 1.

**Table 1:** Patient characteristics.

Factors	Total cohort (N = 292)
Age (years)	48.5 ± 12.5
Sex (female)	228 (78.1%)
Tumor size (cm)	1.37 ± 0.95
Tumor classification (T1/T2/T3)	78/10/204
LN classification (N0 or Nx/N1a/N1b)	41/169/82
Preablation TSH-stimulated serum Tg (ng/mL)	2.62 ± 8.03
Administered RAI activity (mCi) (30/100/150)	24/265/3
Preparation of RAI ablation with rhTSH	264 (90.4%)
2015 ATA risk group (low/intermediate)	61/231

Data are presented as number (percentage) or mean ± standard deviation. ATA: American Thyroid Association; TSH: thyroid-stimulating hormone; LN: lymph node; RAI: radioactive iodine; rhTSH: recombinant human thyroid-stimulating hormone

### Risk factors for RAI-avid metastatic LN in the low- and intermediate-risk groups

**Fig 1:**
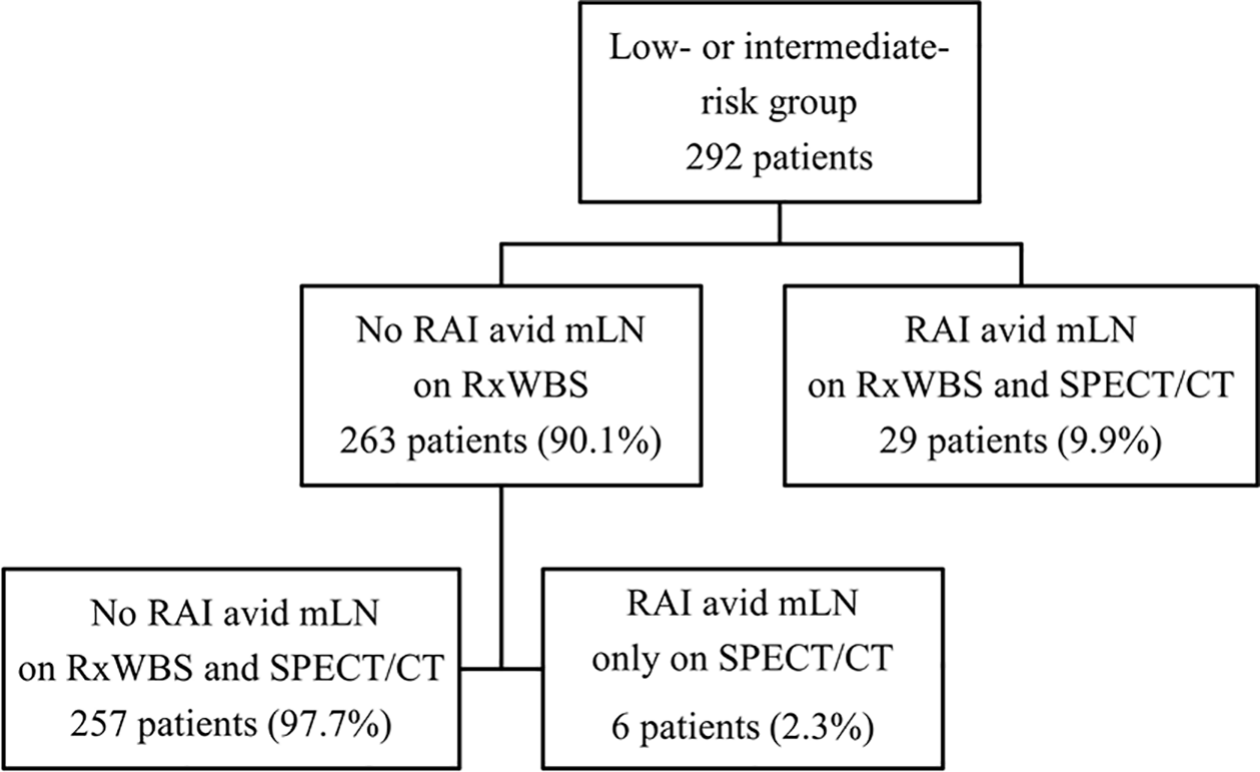
Result of postablation I-131 whole-body scintigraphy and postablation SPECT/CT in the low-or intermediate-risk group. SPECT/CT: single-photon emission computed tomography; RAI: radioactive iodine; mLN: metastatic lymph node; RxWBS: postablation I-131 whole-body planar scintigraphy.

**Fig 2:**
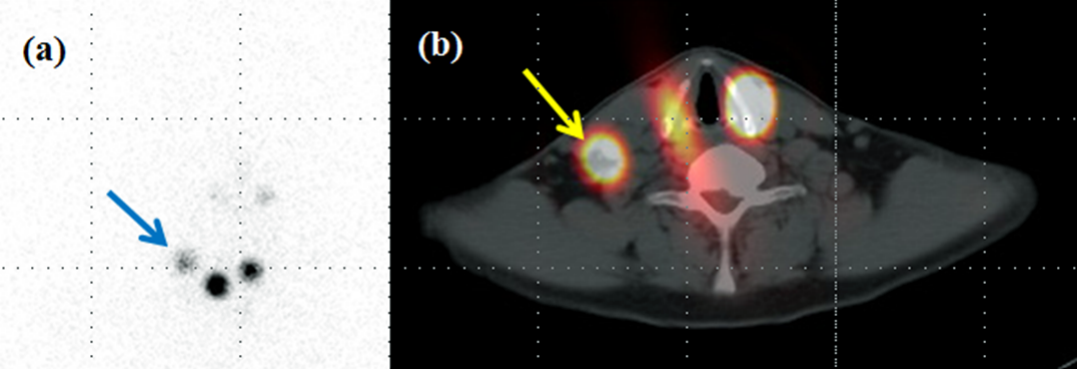
Postablation I-131 whole body planar scan and SPECT/CT of a patient with papillary thyroid cancer. Preablation TSH-stimulated serum thyroglobulin level was 4.7 ng/ml. A focal iodine uptake was seen in the right neck on postablation I-131 planar scintigraphy (a). The SPECT/CT image also revealed RAI uptake on a small LN in the right upper neck (b).

**Fig 3:**
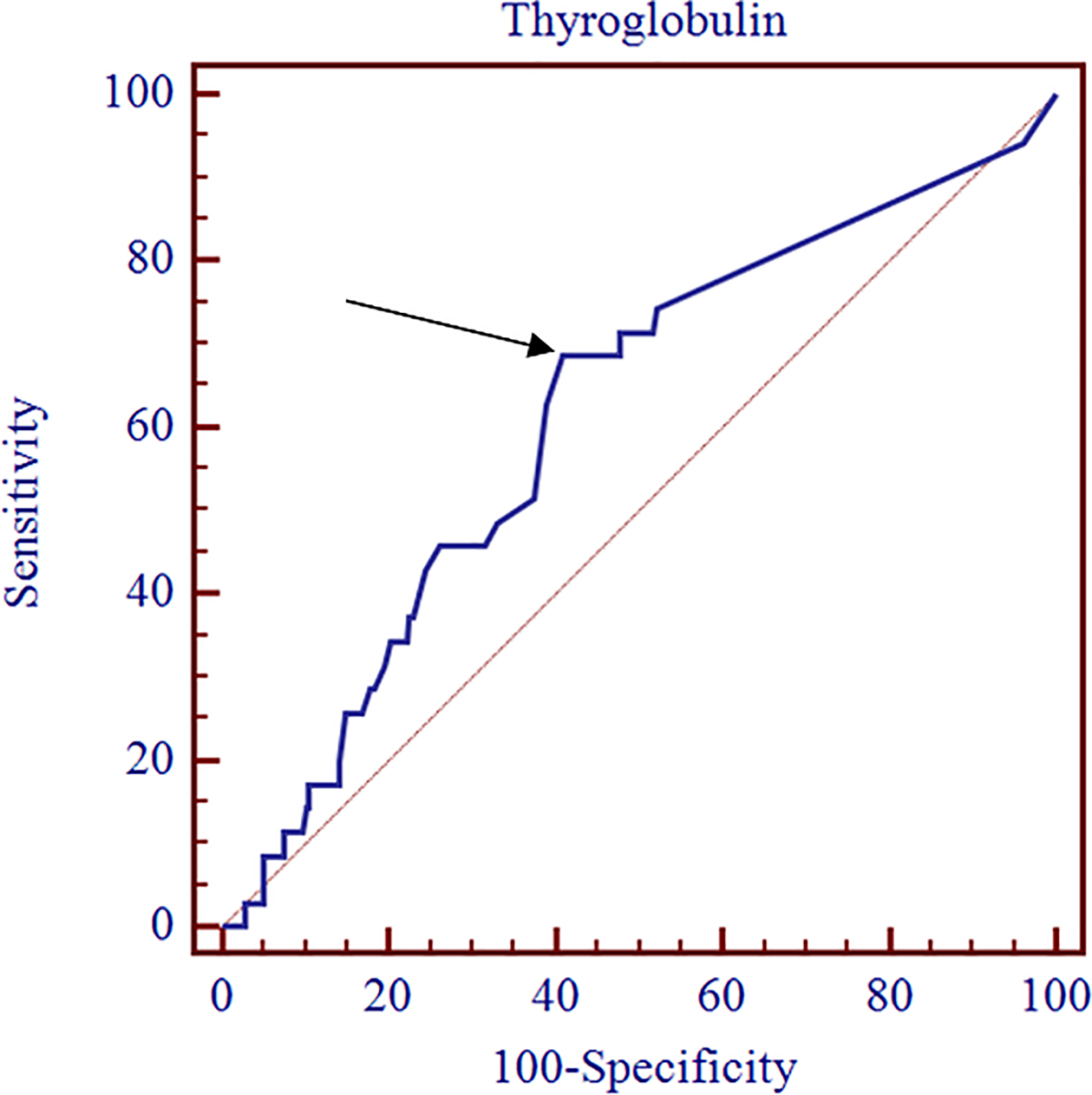
Receiver-operating-characteristics (ROC) curve analysis for preablation TSH-stimulated serum thyroglobulin level of RAI avid metastatic lymph node on SPECT/CT in low- or intermediate-risk group. The optimal cut-off value of TSH-stimulated serum Tg was 0.5 ng/ml for predicting residual RAI avid mLN (sensitivity, 68.6%; specificity, 61.1%; area under the curve, 0.615; p = 0.022).

A RAI-avid mLN was identified on RxSPECT/CT in 35 (12%) of the patients (Fig 1). The clinical characteristics of the 35 patients with RAI-avid mLN on RxSPECT/CT are summarized in Table 2. Meanwhile, no RAI-avid mLN was detected on RxSPECT/CT in 257 (88%) of the patients, similar to the results of RxWBS (Fig 2). However, RxSPECT/CT showed additional RAI-avid mLN that were not revealed on RxWBS in 6 patients (2.3%). No significant differences in the clinicopathological factors, except for preablation TSH-stimulated serum Tg, were found between patients with and without RAI-avid mLN. The optimal cut-off value of preablation TSH-stimulated serum Tg level for predicting residual RAI-avid mLN determined using ROC analysis was 0.5 ng/ml (sensitivity, 68.6%; specificity, 61.1%; p = 0.022; area under the curve, 0.615; Fig 3). In the multivariate analyses, preablation TSH-stimulated serum Tg was still a statistically significant predictor for hidden RAI-avid mLN after adjusting for age, tumor size, and N stage, (cut-off = 0.5; hazard ratio (HR): 2.96; p = 0.04). Among the 146 patients whose TSH-stimulated serum Tg levels were over 0.5 ng/ml, 24 (16.4%) had RAI-avid mLN (Table 3). Meanwhile, among the 146 patients with TSH-stimulated serum Tg levels under 0.5 ng/ml, 11 patients (7.5%) had RAI-avid mLN and all 11 patients were in the intermediate group. In the low-risk group, no patients with TSH-stimulated serum Tg levels under 0.5 ng/ml had RAI-avid mLN.

**Table 2:** Comparison of clinicopathological parameters between patients with and without RAI-avid mLN.

Factors	RAI-avid mLN (+) (N = 35)	RAI-avid mLN (−) (N = 257)	p value
Age (years)	48.0	48.0	0.55
Sex (female/male)	26/9	202/55	0.718
Operation type (cND/mRND)	26/9	199/58	0.977
Tumor size (cm)	1.1	1.1	0.338
Number of mLN (mean ± SD)	4.0	4.0	0.958
Tumor classification (T1/T2/T3)	6/3/26/0/0	72/7/178/0/0	0.102
N stage (Nx or N0/N1a/N1b)	5/20/10	36/149/72	0.67
Preablation TSH-stimulated serum Tg (ng/mL)	0.8	0.31	0.03
ATA risk group (low/intermediate)	4/31	57/200	0.217

Data are presented as number (percentage) or median. cND: central neck dissection, mRND: modified radical neck dissection, TSH: thyroid-stimulating hormone, Tg: thyroglobulin; SD: standard deviation; mLN: metastatic lymph node

**Table 3:** Prevalence of RAI-avid mLN according to risk group and preablation TSH-stimulated serum Tg level.

		Low-risk RAI-avid mLN (+/−)	Intermediate-risk RAI avid-mLN (+/−)
Preablation TSH-stimulated Tg (ng/mL)	< 0.5	0/32	7/114
	≥ 0.5	4/25	24/86

mLN: metastatic lymph node; RAI: radioactive iodine; TSH: thyroid-stimulating hormone

### Risk factors for RAI-avid metastatic LN in the low-risk group

**Fig 4:**
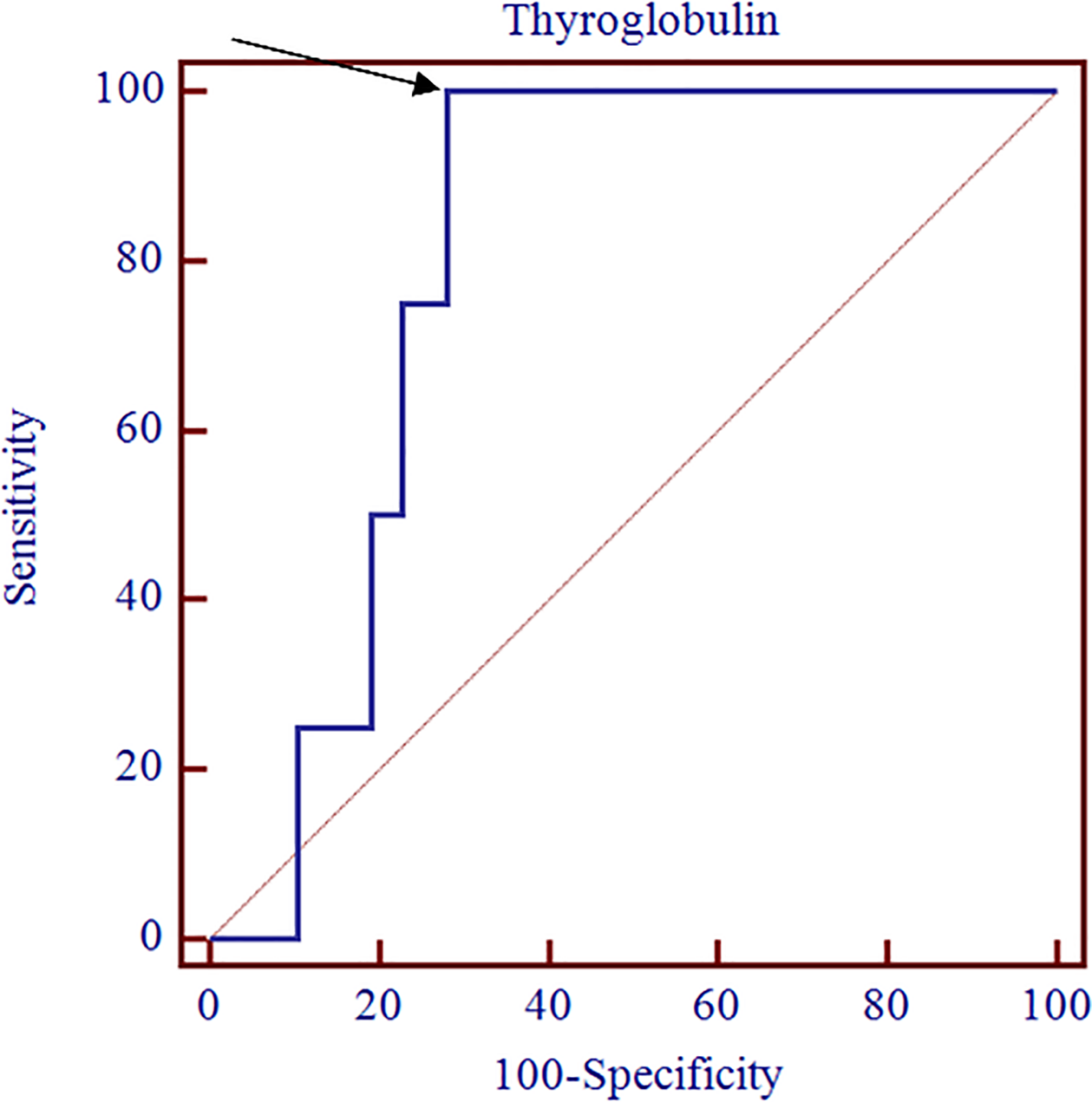
Receiver-operating-characteristics (ROC) curve analysis for preablation TSH-stimulated serum thyroglobulin level of RAI avid metastatic lymph node on SPECT/CT in low-risk group. The optimal cut-off of TSH-stimulated serum Tg level for predicting RAI-avid mLN in the low-risk group was 1.0 ng/ml (sensitivity, 100.0%; specificity, 71.9%; area under the curve, 0.798; p = 0.039).

Four of the 61 patients (6.5%) in the low-risk group showed RAI-avid mLN and were recategorized into the intermediate-risk group due to the presence of mLN. Hidden RAI-avid mLN was predicted only by preablation TSH-stimulated serum Tg among patients in the low-risk group (Table 4). The optimal cut-off value for the capability of preablation TSH-stimulated serum Tg level to predict residual RAI-avid mLN was 1.0 ng/ml in the low-risk group (sensitivity, 100.0%; specificity, 71.9%; area under the curve, 0.798; p = 0.039; Fig 4) as analyzed using ROC curve. In the multivariate analyses, preablation TSH-stimulated serum Tg level was a statistically significant predictor of hidden RAI-avid mLN in the low-risk group after adjusting for age, sex, and N stage (cut-off = 1.0; HR: 5.3; p = 0.03). The lowest value of TSH-stimulated serum Tg among patients with RAI-avid mLN was 0.9 ng/ml.

**Table 4:** Comparison of clinicopathological parameters between patients with and without RAI-avid mLN in the low-risk group.

Factors	RAI-avid mLN (+) (N = 4)	RAI-avid mLN (−) (N = 57)	p value
Tumor size (cm)	1.40	0.80	0.406
Number of mLN	0.50	2.00	0.187
T stage (T1/T2/T3/T4)	3/1/0/0	52/5/0/0	0.853
N stage (Nx or N0/N1a/N1b)	2/2/0	12/45/0	0.371
Preablation TSH-stimulated serum Tg (ng/ml)	1.75	0.24	0.03

Data are presented as number or median. TSH: thyroid-stimulating hormone; Tg: thyroglobulin; mLN: metastatic lymph node; LN: lymph node; RAI: radioactive iodine

### Risk factors for RAI-avid metastatic LN in the intermediate-risk group

In the intermediate-risk group (N = 231 patients), 31 patients (13.0%) had hidden RAI-avid mLN on RxSPECT/CT. Patients with RAI-avid mLN had higher TSH-stimulated serum Tg values than patients without mLN, but the difference was not statistically significant. All clinicopathological data according to mLN status on RxSPECT/CT were not significantly different in this group (Table 5).

**Table 5:** Comparison of clinicopathological parameters between patients with and without RAI-avid mLN in the intermediate-risk group.

Factors	RAI-avid mLN (+) (N = 31)	RAI-avid mLN (−) (N = 200)	p value
Tumor size (cm)	1.10	1.20	0.801
Number of mLN	4	5	0.712
T stage (T1/T2/T3/T4)	3/2/26/0	20/2/178/0	0.096
N stage (Nx or N0/N1a/N1b)	3/18/10	24/104/72	0.197
Preablation TSH-stimulated serum Tg (ng/ml)	0.70	0.35	0.105

Data are presented as number or median. TSH: thyroid-stimulating hormone, Tg: thyroglobulin, mLN: metastatic lymph node; RAI, radioactive iodine

## Discussion

In our study, the incidence of RAI-avid mLN that was newly identified on RxSPECT/CT was relatively high (12%, 35/292) in patients with PTC who belonged to the low- or intermediate-risk groups. Four (6.5%) out of the 61 patients in the low-risk group had postoperative hidden RAI-avid mLN, and their risk status was changed to intermediate after the identification of the mLN. In this study, all subjects received the prophylactic central neck dissection, therefore this result might not be similar to patients who did not receive prophylactic central neck dissection. We think that frequency of LN metastasis could be higher in patients did not received the prophylactic central neck dissection.

Among the clinical and pathological parameters, preablation TSH-stimulated serum Tg level was the sole predictor of the presence of hidden RAI-avid mLN. In the subgroup analysis, preablation TSH-stimulated serum Tg level was the only predictor of the presence of RAI-avid mLN in the low-risk group. However, no significant predictors of RAI-avid mLN were found in the intermediate-risk group.

Many retrospective studies have reported that postoperative TSH-stimulated serum Tg is a useful predictor of persistent/recurrent disease in thyroidectomized patients with thyroid cancer [8–11]. Some studies reported that RAI-avid mLN was seen in 6% of patients with stimulated Tg < 1.5 ng/ml [12, 13]. On the other hand, Giovanella et al. showed no RAI uptake outside the thyroid bed in patients with non-stimulated Tg < 0.4 ng/ml [9]. In our study, the level of preablation TSH-stimulated serum Tg was significantly different between patients with RAI-avid mLN on RxSPECT/CT and patients without mLN. Among the 146 patients with TSH-stimulated serum Tg levels under 0.5 ng/ml, 11 (7.5%) had RAI-avid mLN. However, a direct comparison of our study and the cohort used by Giovanella et al. is complicated by significant differences in enrolled patients and imaging modality of postablation I-131 scintigraphy. The current study included higher-risk patients (70% T3 vs. 10% in the Giovanella cohort, 28% N1b vs. 9% in the Giovanella cohort) and used SPECT/CT technology that is capable of sensitive detection of RAI-avid mLN. Moreover, no patient with TSH-stimulated serum Tg level under 0.5 ng/ml in the low-risk group showed RAI-avid mLN in the current study. One study reported no uptake outside the thyroid bed in 132 low-risk patients with THW Tg level of < 1 ng/ml [14]. The results of the current study is similar to that study; however, in this study, the group with low -risk of recurrence was defined as the lack of LN involvement upon preoperative and perioperative examination by applying the 2009 ATA guidelines for DTC [15], and a postablation planar scan was used [14]. By contrast, patients with fewer than 5 pathologic central neck LN micrometastasis were classified as low-risk according to the 2015 ATA guideline in the current study, and SPECT/CT was applied for post I-131 ablation scintigraphy to detect RAI-avid mLN. Our results demonstrated that preablation TSH-stimulated serum Tg level predicted RAI-avid mLN.

The optimal cut-off value of TSH-stimulated serum Tg for predicting RAI-avid mLN as determined via ROC curve analysis was 0.5 ng/ml in low or intermediate-risk group (sensitivity, 68.6%; specificity, 61.1%; area under the curve, 0.615; p = 0.022; Fig 3) and 1.0 ng/ml in the low-risk group (sensitivity, 100.0%; specificity, 71.9%; area under the curve, 0.798; p = 0.039; Fig 4). In the multivariate analysis, preablation TSH-stimulated serum Tg level was a predictor of RAI-avid mLN (low or -risk group cut-off = 0.5, HR: 2.96, p = 0.04; low-risk group cut-off = 1.0, HR: 5.3, p = 0.03). The lowest value of preablation TSH-stimulated serum Tg in the low-risk patients with RAI-avid mLN was 0.9 ng/ml. Therefore, a high TSH-stimulated serum Tg level can be a valuable indicator for applying RAI ablation to rule out hidden RAI-avid mLN in the low-risk group, and 0.9 or 1.0 ng/ml of TSH-stimulated serum Tg may be a reasonable predictor of RAI-avid mLN in the low-risk group.

In our study, patients with RAI-avid mLN had higher TSH-stimulated serum Tg values than patients without mLN in intermediate-risk group, but the difference was not statistically significant. In 117 patients with TSH-stimulated serum Tg level ≥ 0.5 ng/ml, RAI-avid mLN were observed in 20 patients (17.1%). However only 11 (9.6%) among 114 patients having TSH-stimulated serum Tg levels < 0.5 ng/ml had RAI-avid mLN (Table 3). In low-risk group, all patients having RAI-avid mLN had high TSH-stimulated serum Tg which was a predictor for RAI-avid mLN, however, 11 patients having low TSH-stimulated serum Tg revealed RAI-avid mLN on RxSPECT/CT. In intermediate-risk group, TSH-stimulated serum Tg was not statistically significant factor, because RAI-avid mLN can be exist in patients having low TSH-stimulated serum Tg.

Before the introduction of SPECT/CT technology, planar post I-131 ablation scan had been used to identify RAI-avid metastatic foci. However, differentiating RAI-avid mLN from physiological RAI accumulation and remnant thyroid tissue on planar post I-131 ablation scan might be difficult due to the lack of anatomical detail and the overlap of RAI uptake areas. The combination of SPECT/CT and planar scan provides additional information about RAI-avid metastatic foci [4–6, 16]. Wang et al. reported that SPECT/CT can identify new metastatic lesion missed by planar scan in 7% of patients [17]. Jeong et al. also reported that SPECT/CT found additional mLN in 5.2% of patients with negative findings on planar scan [18]. In our study, SPECT/CT revealed abnormal findings that were not visible on RxWBS in 6 patients (2.2%). Compared with a previous study, the frequency of additional information provided by SPECT/CT is lower in the current study, and this might be related to the higher percentage of low-risk patients in our study (11% low-risk vs. 1% in the Jeong cohort; 89% intermediate-risk vs. 91% in the Jeong cohort; 0% high risk vs 8% in the Jeong cohort). SPECT/CT can provide specific and sensitive information about RAI-avid mLN and facilitate accurate cancer staging that should help in establishing a more appropriate and individualized treatment plan.

Our study had several limitations. First was the retrospective design of our study in which we tried to include patients who underwent surgery and followed up in one hospital to ensure a similar therapeutic approach, imaging studies, and laboratory evaluation. Second, although the low-risk group defined by the 2015 ATA guideline includes clinical N0 or under 5 pathologic N1 micrometastases (< 0.2 cm in the largest dimension), we had limited information regarding the size of mLN. Each patient in the low-risk group is considered to be in the N0 clinical stage, which means that no suspicious LN metastasis was found on preoperative neck US. Even though cervical mLN might be present, it would be too small to be detected on preoperative US, which might mean no suspicious macrometastatic LN. Third, RAI was administered either after rhTSH or after THW, and the TSH-stimulated serum Tg levels might be affected by methods for TSH elevation [19]. However, in the current study, the TSH-stimulated serum Tg levels did not significantly differ according to the type of method for TSH elevation applied in the low-risk group (THW vs. rhTSH: 1.81±3.01 ng/ml vs. 1.86 ±1.99 ng/ml, p = 0.628), or in the intermediate-risk group (THW vs. rhTSH: 2.23±4.18 ng/ml vs. 3.56 ±4.28 ng/ml, p = 0.452). Thus, regardless of the method for TSH elevation, the same cut-off level of Tg was applied. Forth, Remnant thyroid tissue can influence Tg level. The amount of residual thyroid tissue after total thyroidectomy may vary according to surgeons and applied surgical techniques, and it can influence level of TSH-stimulated serum Tg. In the current study, surgery was performed by only two experienced high-volume thyroid surgeons who performed more than 300 cases of thyroid cancer surgery per year. So amount of remnant tissue might not be very different for the subjects. Although 1 ng/ml of TSH-stimulated serum Tg was optimal cut-off value for predicting RAI-avid mLN in this study, the cut off may not be appropriate for other institutions due to possible difference in amount of the remnant tissue. In addition, measured Tg values also can be different by used assay kits. Fifth, if stimulated Tg is available in clinics, we can use the Tg to predict presence of RAI-avid mLN. In this retrospective study, data about unstimulated Tg was not available, which might be more clinically relevant and be feasible to apply. We think that unstimulated Tg might also be one of prognostic factor for predicting the RAI-avid mLN. Additional studies needed to elucidate prognostic value of the unstimulated Tg for radioiodine avid mLN.

In the current study, patient risk was stratified according to the 2015 ATA guideline, and RAI-avid mLN was assessed by SPECT/CT. Our results demonstrated that preablation TSH-stimulated serum Tg can predict hidden RAI-avid mLN, especially in the low-risk group.

## Conclusion

The incidence of RAI-avid mLN on RxSPECT/CT was relatively high in both low- and intermediate-risk patients with PTC, and high preablation TSH-stimulated serum Tg level was a predictor of metastasis, especially in the low-risk group. The results of the current study suggest that the therapeutic plan, including RAI ablation, can be individualized according to preablation TSH-stimulated serum Tg level in low-risk patients with PTC.
